# The methylation-expression correlation of autophagy-related genes in colorectal cancer patients from southern Iran

**DOI:** 10.22099/mbrc.2025.52486.2101

**Published:** 2025

**Authors:** Maryam Niknam, Fakhraddin Naghibalhossaini, Seyed Vahid Hosseini, Mozhdeh Zamani, Pooneh Mokarram

**Affiliations:** 1 Autophagy Research Center, Shiraz University of Medical Sciences, Shiraz, Iran; 2 Department of Biochemistry, School of Medicine, Shiraz University of Medical Sciences, Shiraz, Iran; 3 Colorectal Research center, Shiraz University of Medical Sciences, Shiraz, Iran; 4 Autophagy Research Center, Department of Biochemistry, School of Medicine, Shiraz University of Medical Sciences, Shiraz, Iran

**Keywords:** Promoter methylation, Epigenetics, Gene regulation, Autophagy signaling

## Abstract

Colorectal cancer (CRC), which has high mortality and increasing morbidity is a major concern worldwide. The autophagy pathway plays a crucial role in carcinogenesis and drug resistance in this disease. Epigenetic modification is one of the main regulatory mechanisms for this pathway. This study aimed to investigate the impact of promoter methylation as one of the epigenetic modifications on the expression of autophagy-associated genes (ATGs) (*ATG2B, ATG4D, ATG9A,* and *ATG9B*) in 21 CRC patients from southern Iran. The tissue DNA and RNA were extracted by standard phenol-chloroform extraction method and A BIOZOL RNA isolation kit, respectively. The methylation status and transcript levels of desired genes were ascertained using the methylation-specific PCR and quantitative real-time PCR methods, respectively. In the majority of studied patients, the relative mRNA expressions of *ATGs* were significantly higher in CRC tissues compared to normal ones. There was no significant relationship between the methylation of the *ATG* genes and clinicopathological features of CRC patients. Interestingly, in most of the patients, the promoter hypermethylation of the *ATG2B*, *ATG4D*, *ATG9A* and *ATG9B* genes led to their high mRNA expression. Although promoter hypermethylation usually suppresses gene expression, the cancer type, stage, and compensatory mechanisms may reverse this association. This highlights the complexity of the epigenetic regulation of *ATG2B*, *ATG4D*, *ATG9A* and *ATG9B* genes in CRC. Further large-scale studies will contribute to discovering the exact influences of *ATG* methylation in CRC carcinogenesis and thereby may thereby provide novel targets and biomarkers for this lethal illness.

## INTRODUCTION

As a usual digestive tract tumor, colorectal cancer (CRC) is the third main cause of cancer-associated deaths in the world [1]. Over the past decades, great advances have been made in the prevention, diagnosis, and treatment of cancer, such as personalized medicine, however, the survival rate of CRC patients is still insufficient [2]. Recently, the global burden of CRC will enhance by 60%, to over 1.1 million deaths and 2.2 million new cases by 2030 [3]. An understanding of pathogenesis, and study for new prognostic cancer biomarkers, may affect advancement in cancer prognoses and may also cause the identification of novel promising targets for anticancer therapies. A large body of evidence has revealed autophagy as a potential new biomarker in CRC development [4]. 

As a multistep catabolic pathway, autophagy is a critical process in cancer initiation and development [5]. Autophagy plays paradoxical and complicated roles in cancer via interactions with cell survival, cell metabolic reactions, and the turnover of proteins and organelles at multiple levels [6]. With both promoting and inhibitory effects on cancer cells, autophagy can prevent tumor formation in early stages while it can lead to tumor cell survival and malignant transformation in response to various stressful triggers in later stages of cancer [7, 8]. It has been proposed that multiple signaling cascades, such as epigenetic regulation, have crucial roles in autophagy deregulation. However, the effect of epigenetic regulation on autophagy is largely debatable [9]. 

CRC is a multifactorial disease that arises due to the cumulative accumulation of genetics as well as epigenetic alterations [10]. Epigenetic modifications, which change the expression of important genes related to physiological and pathological processes without influencing the DNA sequence, have been reported to play a critical role in the initiation and progression of various cancers, such as gastrointestinal cancers. Autophagy may be regulated by various epigenetic processes, including DNA methylation, microRNA-associated modulation, and histone modifications [9, 11, 12]. Current studies have associated autophagic flux with epigenetic regulation [13, 14]. The clinical and biological importance of epigenetic regulation of autophagy in cancer has increasingly received vast attention among researchers worldwide. Deviant epigenetic changes of autophagic regulators are considered key determinants of the cancer fate and are evident in multiple ways: promotion or prohibition of autophagy that can be protective or lethal, thereby contributing to tumor suppression, progression, metastasis, or development of chemo- or radio-resistance in established tumors [11]. 

As a highly conserved pathway, autophagy is severely orchestrated by key autophagy proteins, which are encoded by more than thirty autophagy-related genes (*ATG*) [4]. As the main components of the autophagy-mediated regulatory network, ATGs contribute to CRC occurrence and development [15]. ATGs regulate the autophagy pathway and are frequently controlled by epigenetic modifications, including DNA methylation, histone modification, and microRNA-mediated gene regulation [16]. Altered expression (either over- or under-expression) of autophagy genes may be considered a crucial factor in the initiation and progression of numerous cancers [17]. In humans, *ATG* genes are regulated at both transcriptional and post-translational levels, as well as by epigenetic modifications [18]. Based on previous studies, compared with other mentioned epigenetic mechanisms, DNA hypermethylation has a more distinguished role in the regulation of autophagy [19]. Epigenetic silencing of some key genes by DNA methylation is involved in the autophagy regulation in all stages of cancer [9]. 

In melanoma, *ATG5* down-regulation by its promoter hypermethylation is correlated with cell proliferation and tumorigenesis [20]. *ATG16L2* is frequently silenced by DNA methylation, and its inactivation is associated with poorer outcomes than imatinib treatment in hematological cancers [9]. In breast cancer, abnormal methylation of *BECLIN-1*, an autophagy-related gene that acts as a tumor suppressor, can be considered as a mechanism of autophagy prevention and induction of tumor [21-23]. Similarly, promoter hypermethylation of* ATG4D*, *ATG2B*, *ATG9A*, and *ATG9B *genes has been demonstrated in most invasive ductal carcinomas (IDC), and this enhanced methylation was associated with the cancer grade, reduced gene expression, and lymph node metastasis [24].

The activation and biological effects of cellular autophagy are controlled by various autophagy-related genes (ATG) and signaling pathways [25]. Among these pathways, epigenetic modifications greatly contribute to the process of cellular autophagy [11]. The epigenetic control of autophagy is very complicated and has a dual role in CRC. Therefore, it is still controversial whether autophagy activation or inhibition promotes cancer development [7]. Regarding the lack of knowledge on the effect of epigenetic modification of autophagy in CRC, the present study aimed to investigate the impacts of epigenetic alterations of the genes related to autophagy (*ATG2B, ATG4D, ATG9A,* and *ATG9B*) in CRC patients. It will contribute to detecting potential mechanisms that regulate autophagy in disease searching for novel targets for more efficient diagnosis and treatment of CRC. 

## MATERIALS AND METHODS

### Patients and tumor samples:

The surgically resected tumor and normal adjacent tissues (the tissue surrounding the tumor that appears histologically normal but may have molecular alterations) were collected from 21 colorectal cancer patients at one university-related hospital in southern Iran, Shiraz, from 2021 to 2022. Informed consent was received from each patient or the patient’s supervisor. The institutional ethics committee (Ethical Approval ID: IR.SUMS. REC.1402.205) ethically confirmed this study. At once after surgical resection, the cancerous and normal adjacent tissues were snap-frozen and stored at -80ºC. Then, the histological diagnosis was done by an expert pathologist who ascertained the appropriate tissue sections for further extraction of DNA and RNA and molecular analyses. The clinicopathological features of patients were assessed from hospital records. Inclusion criteria were patients diagnosed with colorectal cancer, individuals aged 18 years or older at the time of diagnosis, patients with a pathologically confirmed adenocarcinoma of the colon or rectum, and those able to provide written informed consent for participation in studies. Exclusion criteria were patients diagnosed with other cancers, individuals with a strong family history of colorectal cancer or other relevant cancers, individuals younger than 18 at the time of diagnosis, patients without a pathologically confirmed adenocarcinoma of the colon or rectum, and those who are unable to provide written informed consent for participation in studies.

### DNA extraction:

The canonical proteinase K digestion and phenol-chloroform technique were applied for the extraction of genomic DNA from cancerous and normal adjacent samples [26]. 

### Methylation-specific PCR (MSP) assay of the methylation of gene promoter:

The MSP method was performed for the determination of promoter methylation status of 4 *ATGs *(*ATG9A,* *ATG9B*, *ATG4D*, and *ATG2B*) in normal and tumor tissues [27]. Briefly, genomic DNA (1 µg) was exposed to sodium bisulfite, and then methylated and unmethylated specific primers were utilized for PCR amplification (Table S1). Finally, the obtained products were observed utilizing electrophoresis on 2% agarose gel and UV illumination.

### Gene expression analysis using quantitative Real-Time PCR (qRT-PCR):

A BIOZOL RNA isolation kit (BiofluxBioer, China) was used for total RNA extraction from cancerous and normal adjacent tissues, based on the instructions of the manufacturer. To validate the RNA integrity, 1.5% denaturing agarose gel electrophoresis with 2% formaldehyde was used.

As described previously, the relative expression levels of four *ATGs* (*ATG9A,* *ATG9B*, *ATG4D*, and *ATG2B*) in cancerous and normal adjacent tissues were analyzed by real-time RT-PCR [28]. In brief, the complementary DNA (cDNA) was synthesized based on the manufac-turer’s instructions (Cinagene, Iran). For each studied gene, real-time PCR amplification was performed on 1 µl cDNA in 25 µl reaction mixture using SYBR Green master mix (Ampliqon, Danmark) and gene-specific primer pairs (Table S2) in a QuantStudio™ 3 Real-Time PCR System (Applied Biosystems, USA). The gene amplification was done in triplicate with precycling heat activation at 95°C for 10 min, accompanied by 40 cycles (95°C/15 s, 58°C/30 s, 72°C/30 s, and a final extension at 72°C/10 min). To normalize the levels of gene expression, the 2^-ΔΔCT^ formula was used, and he *β-actin *was also applied as a housekeeping gene. 

### Statistical analyses:

The SPSS version 18 (SPSS Inc., Chicago, IL) was applied for the statistical evaluation. The results are represented as mean±standard deviation (SD). The difference between the two groups was analyzed by an unpaired Student’s *t*-test. Descriptive frequencies and median statistics were applied to evaluate the association between promoter hypermethylation status and mRNA transcript levels for four studied genes. The *p*-value of < 0.05 was considered statistically significant.

## RESULTS

**Figure 1:**
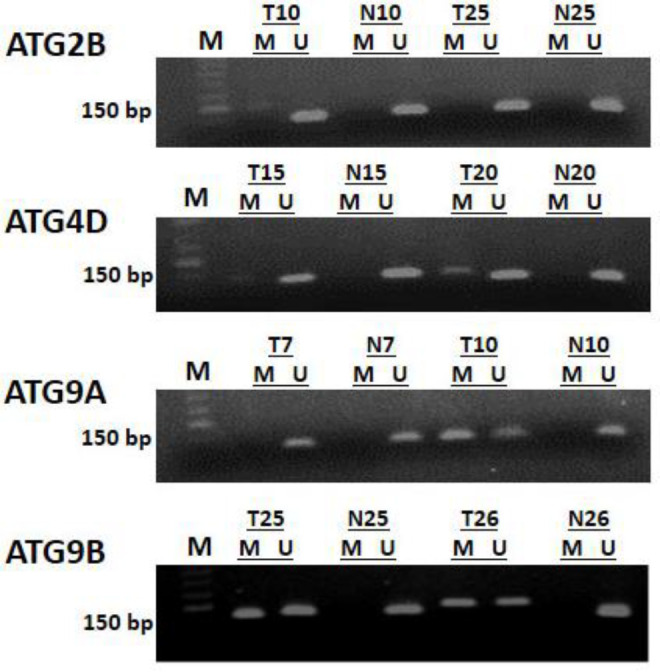
The results of MSP for promoter methylation of *ATG9A,* *ATG9B, ATG4D, *and* ATG2B *genes in colorectal cancer tumors. M: methylated genes; U: unmethylated genes; T: tumor samples, N: Normal adjacent tissues. Lane M demonstrates the 50 bp DNA marker. ATG2B: M=146 bp, U=144 bp; ATG4D: M=157 bp, U=154 bp; ATG9A: M=108 bp, U=108 bp; ATG9B: M=167 bp, U=169 bp.

The clinicopathological characteristics of the patients are indicated in Table 1. Twenty-one (21) patients were included in this study. Most patients were male (66.7%) and older than 60 years (57.1%). Most patients were diagnosed with distal CRC (85.7%, 18 cases) in stages II and III (81%, 17 cases). Most tumors were also well and moderately differentiated (90.5%, 19 cases). As described in the materials and methods section, hypermethylation of CpG islands in tumors was evaluated by the MSP method. The related results are shown in Figure 1.

**Table 1:** Distributions of choosed features of the participants

**Variables**		** n (%)**
**Age**	**< 60 years ** **≥ 60 years**	9 (42.9) 12 (57.1)
**Sex**	**Male** **Female**	14 (66.7) 7 (33.3)
**Stage**	**I** **II** **III**	4 (19) 9 (42.9) 8 (38.1)
**Site**	**Distal** **Proximal**	18 (85.7) 3 (14.3)
**Differentiation**	**Well** **Moderate** **Poor**	13 (61.9) 6 (28.6) 2 (9.5)

The most common methylated locus was *ATG9B* (95.2%; 20 of 21), followed by *ATG4D* (76.2%; 16 of 21), *ATG2B* (57.1%; 12 of 21), and *ATG9A* (47.6%; 10 of 21). Simultaneous promoter hypermethylation of all evaluated genes was detected in six (28.6%) patients. Normal samples were unmethylated for all genes. There was no significant relationship between the *ATG* genes methylation and clinicopathological characteristics of CRC patients (Table 2).

**Table 2:** Gene promoter methylation association with clinicopathological features of colorectal cancerpatients

**Variables**	***ATG2B, n ***				***ATG4D, n***				***ATG9A, n***				***ATG9B, n***		
	M	U	*p*		M	U	*p*		M	U	*p*		M	U	*p*
**Total**	12	9	value		16	5	value		10	11	value		20	1	value
**Age** **<60 (9)** **≥60 (12)**	6 6	3 6	0.66		7 9	2 3	1		3 7	6 5	0.387		8 12	1 0	0.429
**Sex** **Male (14)** **Female (7)**	7 5	7 2	0.642		10 6	4 1	0.624		6 4	8 3	0.659		14 6	0 1	0.074
**Site** **Proximal (3)** **Distal (18)**	0 12	3 6	0.063		2 14	1 4	1		0 10	3 8	0.214		3 17	0 1	1
**Tumor Stage** **I (4)** **II (9)** **III (8)**	1 6 5	3 3 3	0.448		4 6 6	0 3 2	0.670		1 3 6	3 6 2	0.190		4 8 8	0 1 0	1
**Differentiation** **Well (13)** **Moderate (6)** **Poor (2)**	7 3 2	6 3 0	0.650		10 4 2	3 2 0	1		5 4 1	8 2 1	0.659		12 6 2	1 0 0	1

**Figure 2:**
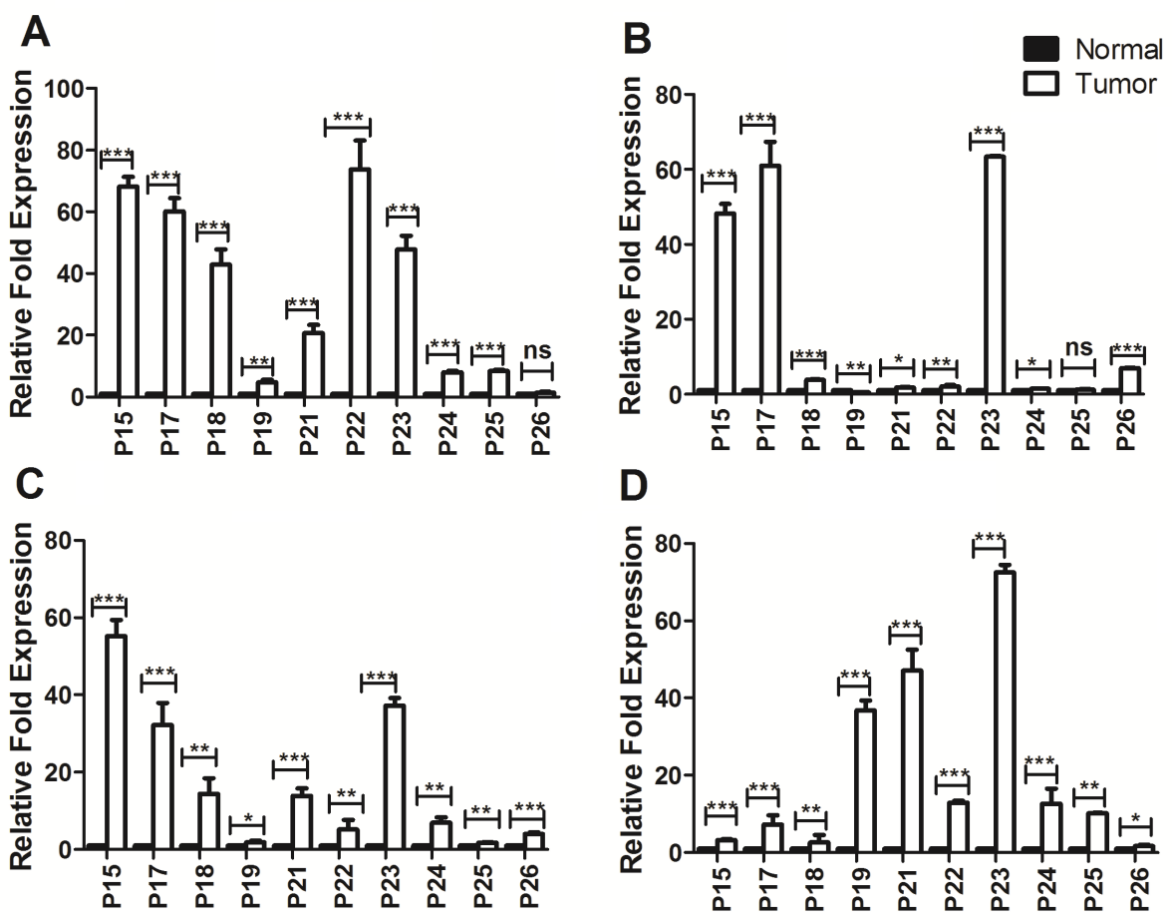
The qRT-PCR analysis of relative expressions of *ATG2B* (A), *ATG4D* (B), *ATG9A* (C), and *ATG9B* (D) in the colorectal cancer tissues (n=10), comparing to the normal counterparts. The transcript levels were normalized using β-actin. The data are indicated as means ± SD of two independent experiments performed in triplicate. (*p<0.05, **p<0.01, and ***p<0.001).

As shown in Figure 2, the expression levels of the *ATG9A,* *ATG9B, ATG4D, *and* ATG2B *genes were analyzed using qRT-PCR. The relative mRNA expressions of *ATG9A*, *ATG9B*, *ATG2B, *and *ATG4D* were significantly greater in CRC tissues compared to normal ones. The upregulation of *ATG2B* and *ATG4D* was just not significant (*p*>0.05) in one evaluated patient (P21 for *ATG4B* and P20 for *ATG4D*). Another exception was observed in P14, which showed a decreased transcript level of *ATG4D* in tumors compared to normal tissues (*p*<0.01).

To determine the promoter methylation of *ATG2B, ATG4D, ATG9A*, and *ATG9B* genes and its impact on their gene expression activity, the transcript level fold changes were compared in methylated and unmethylated CRCs from 10 patients. Our results demonstrated that only in 14.3, 9.5, 19, and 4.7% of cases the promoter hypermethylation of *ATG2B, ATG4D, ATG9A*, and *ATG9B* accompanied by decreased transcript levels of these genes, respectively. However, in other patients, the promoter hypermethylation of studied genes was followed by increased gene expression.

## DISCUSSION

This study shed light on the association between DNA promoter methylation and expression of four ATG genes (*ATG9A,* *ATG9B, ATG4D, *and* ATG2B*) in CRC patients. Our findings challenge the available understanding of promoter hypermethylation as a mechanism of gene silencing, accentuating the complexity of epigenetic regulation in CRC. 

ATG genes play key roles in CRC pathogenesis and therapeutic resistance. *ATG2 *homologs, *ATG2A* and *ATG2B*, are peripheral membrane proteins that contribute to cellular nucleation and the early steps of autophagosome generation. Considerably, the simultaneous silencing of ATG2A and ATG2B leads to autophagy dysfunction and aggregation of autophagic constructs comprising the most of ATG proteins [24, 29]. 

*ATG4 *encodes a member of the ATG4 mammalian family (a group of four cysteine proteases, ATG4A‒D) with endopeptidase function, important for later steps of maturation of autophagosome and its fusion with lysosomes [30]. ATG4D not only exerts an evident role in autophagy regulation but also acts in the interaction between autophagy and apoptosis [31, 32]. Abnormal DNA methylation of *ATG4D* profoundly contributes to various human diseases, including cancers [33, 34]. ATG9 family, with two functional members, ATG9A and ATG9B, is also a multi-transmembrane protein that acts as a membrane transporter in the initial stage of the autophagy flux [35, 36]. ATG9 actively interacts with phagophores but does not convert a stable component of the autophagosome membrane [37]. Loss of *ATG9A* and *ATG9B* genes or disruption of the autophagy pathway is associated with a vast variety of cancers [38]. While promoter hypermethylation generally suppresses gene expression by blocking transcription factor binding or RNA polymerase recruitment, in the current study, we unexpectedly observed an association between promoter hypermethylation of *ATG9A*, *ATG9B*, *ATG2B*, and *ATG4D* genes and their increased expression in the majority of CRC cases.

On the contrary to our findings, in a previous study on breast tumors, promoter hypermethylation of *ATG9A*, *ATG9B*, *ATG2B*, and *ATG4D* genes was linked to reduced gene expression [24].

In parallel with our findings, it has been reported that the hypermethylation of the ZAR1 gene was also accompanied by its upregulation in neuroblastoma [39]. Another study on a PCa mouse model also revealed that the elevated expression level of the GSC (Goosecoid) was related to DNA methylation [40]. A study by Niknam et al. also found that hTERT promoter methylation is directly associated with gene expression that could be explained by the lack of methylation near the transcription start site of hTERT [41].

This paradoxical relationship highlights that epigenetic regulation has a context-dependent nature and may depend on factors such as tumor type, microenvironment, and compensatory pathways [42]. For instance, it has been found that specified genes possessing unmethylated CpG islands in their promoters are unable to generate efficient transcripts as a result of the lack of RNA Pol II recruitment [43]. In addition, local methylation of distinctive residues has been revealed to be critical for the regulation of gene expression and is thus able to counteract the methylation status of the genomic region as a whole [44, 45]. Furthermore, varieties of transcription factors favor binding methylated CpGs rather than unmethylated ones [46-49]. Both sparse and dense methylation of the promoter can hinder the binding of the transcriptional machinery and thereby repress transcription of the related genes. Although the presence of enhancers cannot reactivate the gene expression in densely methylated promoters, enhancers such as SV40 can overcome the sparse methylation and reactivate the gene expression [44, 50, 51]. Nonetheless, it is crucial to notice that there is little data concerning possible mechanisms of gene activation via hypermethylation of DNA and what type of proteins are recruited in these mechanisms.

The present study confirms that a significant fraction of the DNA methylation phenomenon in CRC can be associated with alterations in gene expression levels positively. This highlights the importance of epigenetic evaluations accompanied by analysis of gene expression modifications to define the real impact of promoter methylation on gene transcription activation.

There are some limitations and future directions of this study. The small sample size of 21 patients limited the generalizability of the findings. More multi center studies with larger sample sizes are required to validate these results. Furthermore, it is needed to elucidate the mechanisms by which promoter methylation influences gene expression activation in CRC. In this regard, identifying the transcription factors and enhancers involved in this process could lead to deeper insight into the regulation of *ATG* genes in CRC and thereby may introduce novel targets and biomarkers for this cancer.

In conclusion, this study highlights the complex relationship between promoter methylation and expression of *ATG* genes in CRC. In contrast to the conventional insight, that promoter hypermethylation leads to gene silencing, the current findings indicated expression enhance-ment of *ATG* genes in CRC following promoter hypermethylation in a context-dependent manner. This underscores the complexity of epigenetic regulation in cancer and shows the need for further investigations to fully understand its role in CRC pathogenesis. These insights pave the way for the development of novel biomarkers, therapeutic strategies, and ultimately CRC outcome improvement.

## References

[1] Chisanga D, Keerthikumar S, Pathan M, Ariyaratne D, Kalra H, Boukouris S, Mathew NA, Al Saffar H, Gangoda L, Ang CS, Sieber OM, Mariadason JM, Dasgupta R, Chilamkurti N, Mathivanan S (2016). Colorectal cancer atlas: An integrative resource for genomic and proteomic annotations from colorectal cancer cell lines and tissues. Nucleic Acids Res.

[2] Gil J, Ramsey D, Pawlowski P, Szmida E, Leszczynski P, Bebenek M, Sasiadek MM (2018). The influence of tumor microenvironment on ATG4D gene expression in colorectal cancer patients. Med Oncol.

[3] Sawicki T, Ruszkowska M, Danielewicz A, Niedźwiedzka E, Arłukowicz T, Przybyłowicz KE (2021). A review of colorectal cancer in terms of epidemiology, risk factors, development, symptoms and diagnosis. Cancers (Basel).

[4] Gil J, Pesz KA, Sąsiadek MM (2016). May autophagy be a novel biomarker and antitumor target in colorectal cancer?. Biomark Med.

[5] Hasan A, Haque E, Hameed R, Maier PN, Irfan S, Kamil M, Nazir A, Mir SS (2020). Hsp90 inhibitor gedunin causes apoptosis in A549 lung cancer cells by disrupting Hsp90:Beclin-1:Bcl-2 interaction and downregulating autophagy. Life Sci.

[6] Kimmelman AC (2011). The dynamic nature of autophagy in cancer. Genes Dev.

[7] Galluzzi L, Pietrocola F, Bravo-San Pedro JM, Amaravadi RK, Baehrecke EH, Cecconi F, Codogno P, Debnath J, Gewirtz DA, Karantza V, Kimmelman A, Kumar S, Levine B, Maiuri MC, Martin SJ, Penninger J, Piacentini M, Rubinsztein DC, Simon HU, Simonsen A, Thorburn AM, Velasco G, Ryan KM, Kroemer G (2015). Autophagy in malignant transforma-tion and cancer progression. EMBO J.

[8] Poillet-Perez L, Xie X, Zhan L, Yang Y, Sharp DW, Hu ZS, Su X, Maganti A, Jiang C, Lu W, Zheng H, Bosenberg MW, Mehnert JM, Guo JY, Lattime E, Rabinowitz JD, White E (2018). Autophagy maintains tumour growth through circulating arginine. Nature.

[9] Sui X, Zhu J, Zhou J, Wang X, Li D, Han W, Fang Y, Pan H (2015). Epigenetic modifications as regulatory elements of autophagy in cancer. Cancer Lett.

[10] Sameer AS, Nissar S (2016). Epigenetics in diagnosis of colorectal cancer. Mol Biol Res Commun.

[11] Bhol CS, Panigrahi DP, Praharaj PP, Mahapatra KK, Patra S, Mishra SR, Behera BP, Bhutia SK (2020). Epigenetic modifications of autophagy in cancer and cancer therapeutics. Semin Cancer Biol.

[12] Peixoto P, Grandvallet C, Feugeas JP, Guittaut M, Hervouet E (2019). Epigenetic control of autophagy in cancer cells: A key process for cancer-related phenotypes. Cells.

[13] Lee M, Nam HY, Kang HB, Lee WH, Lee GH, Sung GJ, Han MW, Cho KJ, Chang EJ, Choi KC, Kim SW, Kim SY (2021). Epigenetic regulation of p62/SQSTM1 overcomes the radioresistance of head and neck cancer cells via autophagy-dependent senescence induction. Cell Death Dis.

[14] Zehender A, Li YN, Lin NY, Stefanica A, Nüchel J, Chen CW, Hsu HH, Zhu H, Ding X, Huang J, Shen L, Györfi AH, Soare A, Rauber S, Bergmann C, Ramming A, Plomann M, Eckes B, Schett G, Distler JHW (2021). TGFβ promotes fibrosis by MYST1-dependent epigenetic regulation of autophagy. Nat Commun.

[15] Hao H, Xia G, Wang C, Zhong F, Liu L, Zhang D (2017). miR-106a suppresses tumor cells death in colorectal cancer through targeting ATG7. Med Mol Morphol.

[16] Hu LF (2019). Epigenetic Regulation of Autophagy. Adv Exp Med Biol.

[17] Saha S, Panigrahi DP, Patil S, Bhutia SK (2018). Autophagy in health and disease: A comprehensive review. Biomed Pharmacother.

[18] Peeters JGC, Picavet LW, Coenen SGJM, Mauthe M, Vervoort SJ, Mocholi E, de Heus C, Klumperman J, Vastert SJ, Reggiori F, Coffer PJ, Mokry M, van Loosdregt J (2019). Transcriptional and epigenetic profiling of nutrient-deprived cells to identify novel regulators of autophagy. Autophagy.

[19] Talebian S, Daghagh H, Yousefi B, Ȍzkul Y, Ilkhani K, Seif F, Alivand MR (2020). The role of epigenetics and non-coding RNAs in autophagy: A new perspective for thorough understanding. Mech Ageing Dev.

[20] Liu H, He Z, von Rütte T, Yousefi S, Hunger RE, Simon HU (2013). Down-regulation of autophagy-related protein 5 (ATG5) contributes to the pathogenesis of early-stage cutaneous melanoma. Sci Transl Med.

[21] Khalil H, Tazi M, Caution K, Ahmed A, Kanneganti A, Assani K, Kopp B, Marsh C, Dakhlallah D, Amer AO (2016). Aging is associated with hypermethylation of autophagy genes in macrophages. Epigenetics.

[22] Li Z, Chen B, Wu Y, Jin F, Xia Y, Liu X (2010). Genetic and epigenetic silencing of the beclin 1 gene in sporadic breast tumors. BMC Cancer.

[23] Liang C, Feng P, Ku B, Dotan I, Canaani D, Oh BH, Jung JU (2006). Autophagic and tumour suppressor activity of a novel Beclin1-binding protein UVRAG. Nat Cell Biol.

[24] Zhang X, Li C, Wang D, Chen Q, Li CL, Li HJ (2016). Aberrant methylation of ATG2B, ATG4D, ATG9A and ATG9B CpG island promoter is associated with decreased mRNA expression in sporadic breast carcinoma. Gene.

[25] Li X, He S, Ma B (2020). Autophagy and autophagy-related proteins in cancer. Mol Cancer.

[26] Mokarram P, Naghibalhossaini F, Saberi Firoozi M, Hosseini SV, Izadpanah A, Salahi H, Malek-Hosseini SA, Talei A, Mojallal M (2008). Methylenetetrahydrofolate reductase C677T genotype affects promoter methylation of tumor-specific genes in sporadic colorectal cancer through an interaction with folate/vitamin B12 status. World J Gastroenterol.

[27] Sarabi MM, Naghibalhossaini F (2015). Association of DNA methyltransferases expression with global and gene-specific DNA methylation in colorectal cancer cells. Cell Biochem Funct.

[28] Tatar M, Varedi M, Naghibalhossaini F (2022). Epigenetic effects of blackberry extract on human colorectal cancer cells. Nutr Cancer.

[29] Mandhair HK, Novak U, Radpour R (2021). Epigenetic regulation of autophagy: A key modification in cancer cells and cancer stem cells. World J Stem Cells.

[30] Andaloussi AE, Habib S, Soylemes G, Laknaur A, Elhusseini H, Al-Hendy A, Ismail N (2017). Defective expression of ATG4D abrogates autophagy and promotes growth in human uterine fibroids. Cell Death Discovery.

[31] Kusama Y, Sato K, Kimura N, Mitamura J, Ohdaira H, Yoshida K (2009). Comprehensive analysis of expression pattern and promoter regulation of human autophagy-related genes. Apoptosis.

[32] Li M, Hou Y, Wang J, Chen X, Shao ZM, Yin XM (2011). Kinetics comparisons of mammalian Atg4 homologues indicate selective preferences toward diverse Atg8 substrates. J Biol Chem.

[33] Betin VM, Lane JD (2009). Caspase cleavage of Atg4D stimulates GABARAP-L1 processing and triggers mitochondrial targeting and apoptosis. J Cell Sci.

[34] Kyöstilä K, Syrjä P, Jagannathan V, Chandrasekar G, Jokinen TS, Seppälä EH, Becker D, Drögemüller M, Dietschi E, Drögemüller C, Lang J, Steffen F, Rohdin C, Jäderlund KH, Lappalainen AK, Hahn K, Wohlsein P, Baumgärtner W, Henke D, Oevermann A, Kere J, Lohi H, Leeb T (2015). A missense change in the ATG4D gene links aberrant autophagy to a neurodegenerative vacuolar storage disease. PLoS Genet.

[35] Kojima T, Yamada T, Akaishi R, Furuta I, Saitoh T, Nakabayashi K, Nakayama KI, Nakayama K, Akira S, Minakami H (2015). Role of the Atg9a gene in intrauterine growth and survival of fetal mice. Reprod Biol.

[36] Yamamoto H, Kakuta S, Watanabe TM, Kitamura A, Sekito T, Kondo-Kakuta C, Ichikawa R, Kinjo M, Ohsumi Y (2012). Atg9 vesicles are an important membrane source during early steps of autophagosome formation. J Cell Biol.

[37] Orsi A, Razi M, Dooley HC, Robinson D, Weston AE, Collinson LM, Tooze SA (2012). Dynamic and transient interactions of Atg9 with autophagosomes, but not membrane integration, are required for autophagy. Mol Biol Cell.

[38] Zavodszky E, Vicinanza M, Rubinsztein DC (2013). Biology and trafficking of ATG9 and ATG16L1, two proteins that regulate autophagosome formation. FEBS Lett.

[39] Rauluseviciute I, Drabløs F, Rye MB (2020). DNA hypermethylation associated with upregulated gene expression in prostate cancer demonstrates the diversity of epigenetic regulation. BMC Med Genomics.

[40] Camoriano M, Kinney SR, Moser MT, Foster BA, Mohler JL, Trump DL, Karpf AR, Smiraglia DJ (2008). Phenotype-specific CpG island methylation events in a murine model of prostate cancer. Cancer Res.

[41] Niknam M, Naghibalhossaini F, Zamani M, Hosseini SV, Mokarram P (2024). The effects of thymidylate synthase 3'UTR genotype on methylation of tumor-specific genes promoter in 22 colorectal cancer patients from southern Iran. Mol Biol Res Commun.

[42] Smith J, Sen S, Weeks RJ, Eccles MR, Chatterjee A (2020). Promoter DNA hypermethylation and paradoxical gene activation. Trends Cancer.

[43] Siegfried Z, Simon I (2010). DNA methylation and gene expression. Wiley Interdiscip Rev Syst Biol Med.

[44] Medvedeva YA, Khamis AM, Kulakovskiy IV, Ba-Alawi W, Bhuyan MS, Kawaji H, Lassmann T, Harbers M, Forrest AR, Bajic VB (2014). FANTOM consortium Effects of cytosine methylation on transcription factor binding sites. BMC Genomics.

[45] Fürst RW, Kliem H, Meyer HH, Ulbrich SE (2012). A differentially methylated single CpG-site is correlated with estrogen receptor alpha transcription. J Steroid Biochem Mol Biol.

[46] Zhu H, Wang G, Qian J (2016). Transcription factors as readers and effectors of DNA methylation. Nat Rev Genet.

[47] Yin Y, Morgunova E, Jolma A, Kaasinen E, Sahu B, Khund-Sayeed S, Das PK, Kivioja T, Dave K, Zhong F, Nitta KR, Taipale M, Popov A, Ginno PA, Domcke S, Yan J, Schübeler D, Vinson C, Taipale J (2017). Impact of cytosine methylation on DNA binding specificities of human transcription factors. Science.

[48] Hu S, Wan J, Su Y, Song Q, Zeng Y, Nguyen HN, Shin J, Cox E, Rho HS, Woodard C, Xia S, Liu S, Lyu H, Ming GL, Wade H, Song H, Qian J, Zhu H (2013). DNA methylation presents distinct binding sites for human transcription factors. Elife.

[49] Rishi V, Bhattacharya P, Chatterjee R, Rozenberg J, Zhao J, Glass K, Fitzgerald P, Vinson C (2010). CpG methylation of half-CRE sequences creates C/EBPalpha binding sites that activate some tissue-specific genes. Proc Natl Acad Sci USA.

[50] Weber M, Hellmann I, Stadler MB, Ramos L, Pääbo S, Rebhan M, Schübeler D (2007). Distribution, silencing potential and evolutionary impact of promoter DNA methylation in the human genome. Nat Genet.

[51] Boyes J, Bird A (1992). Repression of genes by DNA methylation depends on CpG density and promoter strength: evidence for involvement of a methyl-CpG binding protein. EMBO J.

