# Supplementary material for: The methylation-expression correlation of autophagy-related genes in colorectal cancer patients from southern Iran
Source: Mol Biol Res Commun. 2025;14(4):307–16. doi: 10.22099/mbrc.2025.52486.2101 (PMC12426958; doi:10.22099/mbrc.2025.52486.2101)
Supplement: Supplementary file 1 — Supplementary Tables S1-S2 [file mbrc-14-307-s001.pdf]

Table S1: Sequences of the methylated (M) and unmethylated (U) specific primers

Gene	Forward primer (5′–3′)	Reverse primer (5′–3′)	Annealing T (°C)	Product size (bp)
ATG2B	U:TAAATTGTTAGGTAAATGGTAATGA<br>M:TGTAAATCGTTAGGTAAACGGTAAC	U:TAAAAAAACTAAAACCCTTCTCAAA<br>M:TAAAAAAACTAAAACCCTTCTCGAA	60	144
ATG4D	U:GGAAATTGAGGTATAGAGAGGTGT<br>M:GAGGAAATTGAGGTATAGAGAGGC	U:ACACCCAAATAAAACTAACTACACA<br>M:AACGCCCAAATAAAACTAACTACG	60	146
ATG9A	U:AATTTTTGTTATTATGGAGTAGTGA<br>M:AATTTTCGTTATTACGGAGTAGCG	U:AAACAAATCTTAAACAATCAACAAC<br>M:AAACGAATCTTAAACAATCAACGAC	61	154
ATG9B	U:TAGATAGGTGATTGAGAGTTGGGAT<br>M:AGATAGGTGATTGAGAGTTGGGAC	U:CCATCAAACCACAACTTCTCCA<br>M:CATCAAACCACGACTTCTCCA	54	157

Table S2: Sequences of primers used for real time PCR

Gene	Forward primer (5′–3′)	Reverse primer (5′–3′)	Annealing T (°C)	Product size (bp)
β-Actin	AATCGTGCGTGACATTAAG	GAAGGAAGGCTGGAAGAG	58	178
ATG2B	CTTCAGATGGAGTTGGAGGAGAC	AGTGGCTCCTTTCAGTCCTACG	58	122
ATG4D	GTGGTGTACGTTTCTCAGGACTG	CACCAGGATGACCACAGACTTC	58	105
ATG9A	GCTTCCTCAAGGAGCAGGTTCA	CCACATTTGCGATAAGGCTCAGG	58	141
ATG9B	ACCCTGTCAGATGCCATCCTAC	CCAGTAGCTGAAGAGGTTGCAG	60	144
